# Supplementary material for: Oestrogen Receptor Isoforms May Represent a Therapeutic Target in Oesophageal Adenocarcinoma
Source: Cancers (Basel). 2022 Apr 8;14(8):1891. doi: 10.3390/cancers14081891 (PMC9032750; doi:10.3390/cancers14081891)
Supplement: Supplementary file 1 [file cancers-14-01891-s001.zip › cancers-1630144-supplementary.pdf]

cancers

Supplementary Files

Oestrogen Receptor Isoforms May Represent a Therapeutic Target in Oesophageal Adenocarcinoma

Steven L. Due, David I. Watson, Isabell Bastian, Ann-Kathrin Eichelmann and Damian J. Hussey

Table S1. Characteristics of cell lines.

Cell line	Est.	Patient	Tumour	Grade	Stage	Doubling Time	Growth Pattern	Reference
Eso-26	2000	56 M	Type I gastro-oesophageal junction adenocarcinoma	-	T4 N1 M1	35-50 h	Loosely adherent spherical clumps in suspension	[1]
Eso-51	2000	74 M	Adenocarcinoma of distal oesophagus, arising in Barrett's	-	IV	-	Spherical clumps in suspension	[1]
Flo-1	1991	68 M	Adenocarcinoma of distal oesophagus	-	-	40-50 h	Adherent monolayer	[2]
JH-EsoAd1	2008	63 M	Adenocarcinoma of distal oesophagus, arising in Barrett's	High	T3 N0 M0	29 h	Adherent monolayer	[3]
OACP4 C	1996	55 M	Gastro-oesophageal junction adenocarcinoma	-	IV	35-50 h	Adherent monolayer	[4]
OE-19	1993	72 M	Primary gastro-oesophageal junction adenocarcinoma	Moderate	III	50-60 h	Adherent in islands	[5]
OE-33	1993	73 F	Adenocarcinoma of distal oesophagus, arising in Barrett's	High	IIA	30-33 h	Adherent monolayer	[5]
SK-GT-4	1989	89 M	Adenocarcinoma of distal oesophagus, arising in Barrett's	Low	T2 N1 M0	30-39 h	Adherent monolayer	[6]
MCF-7	1970	69 F	Metastatic invasive ductal carcinoma of breast (derived from pleural effusion)	-	IV	30-72 h	Adherent monolayer	[7]

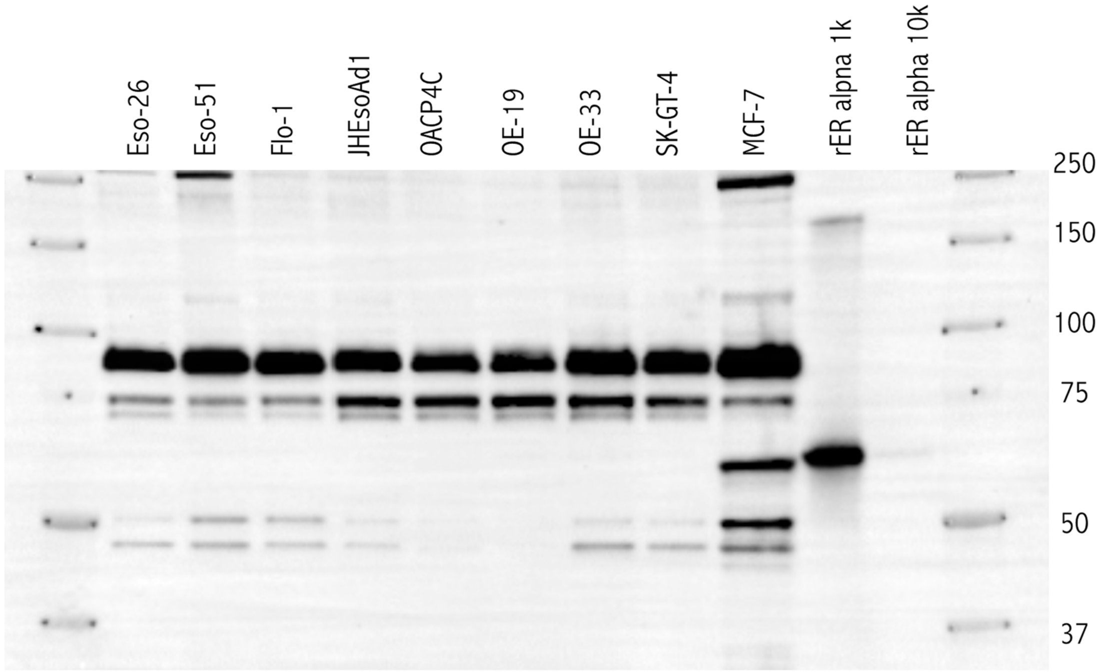

Figure S1. ERα Western blot image presented in Figure 1A.

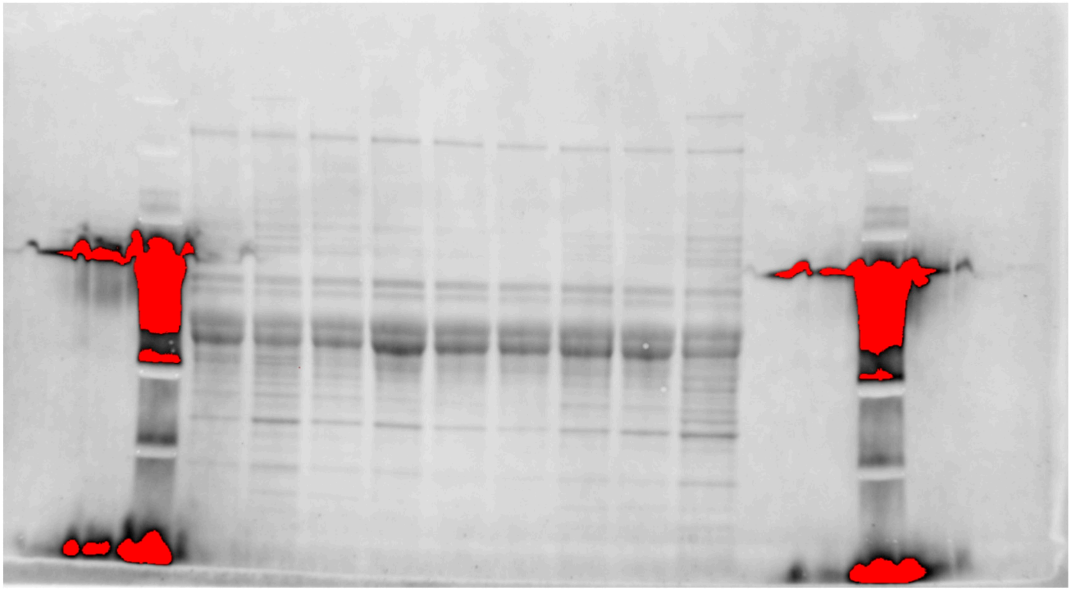

Figure S2. Total protein load image associated with Figure S1.

Table S2. Quantification data associated with ERα blot presented in Figure 1A.

ERα western blot quantification

Protein load normalisation

Serial Number	Net Intensity	Sum Intensity	Mean Intensity	Bkgnd. Intensity	Interior Area (pixels)	**Cell line**	**Normalisation Factor**
1	2625793.75	310613568	62497.6992	61969.3711	4970	Eso-26	1.88381356

2	1882849.63	309870624	62348.2109	61969.3711	4970	Eso-51	1.25522788
3	1207041.63	309194816	62212.2383	61969.3711	4970	Flo-1	0.7560311
4	1610113.63	309597888	62293.3398	61969.3711	4970	JHEsoAd1	1.04622909
5	994977.688	308982752	62169.5703	61969.3711	4970	OACP4C	0.61159953
6	1072673.63	309060448	62185.2031	61969.3711	4970	OE-19	0.66388768
7	1527265.63	309515040	62276.668	61969.3711	4970	OE-33	0.98482186
8	1462177.63	309449952	62263.5742	61969.3711	4970	SK-GT-4	0.93723193

Quantification

**ER alpha 90 kDa species**							
Serial Number	ROI Type	Net Intensity	Sum Intensity	Mean Intensity	Interior Area (pixels)	**Cell line**	**Normalised expression**
1	Rect	3406329.25	7689244	20024.0723	384	Eso-26	1808209.32
2	Rect	4694170	8977085	23377.8262	384	Eso-51	3739695.47
3	Rect	4028274.25	8311189	21643.7207	384	Flo-1	5328185.89
4	Rect	2227449.25	6510364	16954.0723	384	JHEsoAd1	2129026.3
5	Rect	1436692.25	5719607	14894.8105	384	OACP4C	2349073.49
6	Rect	1180249.25	5463164	14226.9902	384	OE-19	1777784.55
7	Rect	3406708.25	7689623	20025.0605	384	OE-33	3459212.65
8	Rect	2418823.25	6701738	17452.4434	384	SK-GT-4	2580816.08
**ER alpha 74 kDa species**							
Serial Number	ROI Type	Net Intensity	Sum Intensity	Mean Intensity	Interior Area (pixels)	**Cell line**	**Normalised expression**
10	Rect	164155.875	1904090	12205.7051	156	Eso-26	87140.1917
11	Rect	157300.375	1897234.5	12161.7598	156	Eso-51	125316.19
12	Rect	153451.625	1893385.75	12137.0879	156	Flo-1	202969.99
13	Rect	462642.875	2202577	14119.084	156	JHEsoAd1	442200.354

14	Rect	502404.125	2242338.25	14373.9629	156	OACP4C	821459.3
15	Rect	558389.875	2298324	14732.8457	156	OE-19	841090.889
16	Rect	521005.375	2260939.5	14493.2012	156	OE-33	529035.142
17	Rect	363607.875	2103542	13484.2441	156	SK-GT-4	387959.332
**ER alpha 70 kDa species**							
Serial Number	ROI Type	Net Intensity	Sum Intensity	Mean Intensity	Interior Area (pixels)	**Cell line**	**Normalised expression**
19	Rect	51040.6328	1790974.75	11480.6074	156	Eso-26	27094.3122
20	Rect	36437.6328	1776371.75	11386.998	156	Eso-51	29028.6995
21	Rect	25030.6328	1764964.75	11313.877	156	Flo-1	33107.9406
22	Rect	97506.7578	1837440.88	11778.4668	156	JHEsoAd1	93198.2857
23	Rect	82190.6328	1822124.75	11680.2871	156	OACP4C	134386.356
24	Rect	109830.133	1849764.25	11857.4629	156	OE-19	165434.812
25	Rect	108163.508	1848097.63	11846.7793	156	OE-33	109830.531
26	Rect	69679.0078	1809613.13	11600.084	156	SK-GT-4	74345.5331
**ER alpha 50 kDa species**							
Serial Number	ROI Type	Net Intensity	Sum Intensity	Mean Intensity	Interior Area (pixels)	**Cell line**	**Normalised expression**
28	Rect	9666.50781	1749600.63	11215.3887	156	Eso-26	5131.35058
29	Rect	72395.6328	1812329.75	11617.498	156	Eso-51	57675.2909
30	Rect	49307.0078	1789241.13	11469.4941	156	Flo-1	65218.2267
31	Rect	10602.5078	1750536.63	11221.3887	156	JHEsoAd1	10134.0212
32	Rect	894.007813	1740828.13	11159.1543	156	OACP4C	1461.75359
33	Rect	-767.24219	1739166.88	11148.5059	156	OE-19	-1155.6807
34	Rect	10464.7578	1750398.88	11220.5059	156	OE-33	10626.0413
35	Rect	11924.8828	1751859	11229.8652	156	SK-GT-4	12723.5131
**ER alpha 46 kDa species**							

Serial Number	ROI Type	Net Intensity	Sum Intensity	Mean Intensity	Interior Area (pixels)	**Cell line**	**Normalised expression**
37	Rect	47254.6328	1787188.75	11456.3379	156	Eso-26	25084.5592
38	Rect	80911.0078	1820845.13	11672.084	156	Eso-51	64459.2184
39	Rect	38171.2578	1778105.38	11398.1113	156	Flo-1	50489.0046
40	Rect	14278.2578	1754212.38	11244.9512	156	JHEsoAd1	13647.3531
41	Rect	341.257813	1740275.38	11155.6113	156	OACP4C	557.97592
42	Rect	2503.25781	1742437.38	11169.4707	156	OE-19	3770.60443
43	Rect	75583.2578	1815517.38	11637.9316	156	OE-33	76748.1517
44	Rect	54632.8828	1794567	11503.6348	156	SK-GT-4	58291.7427

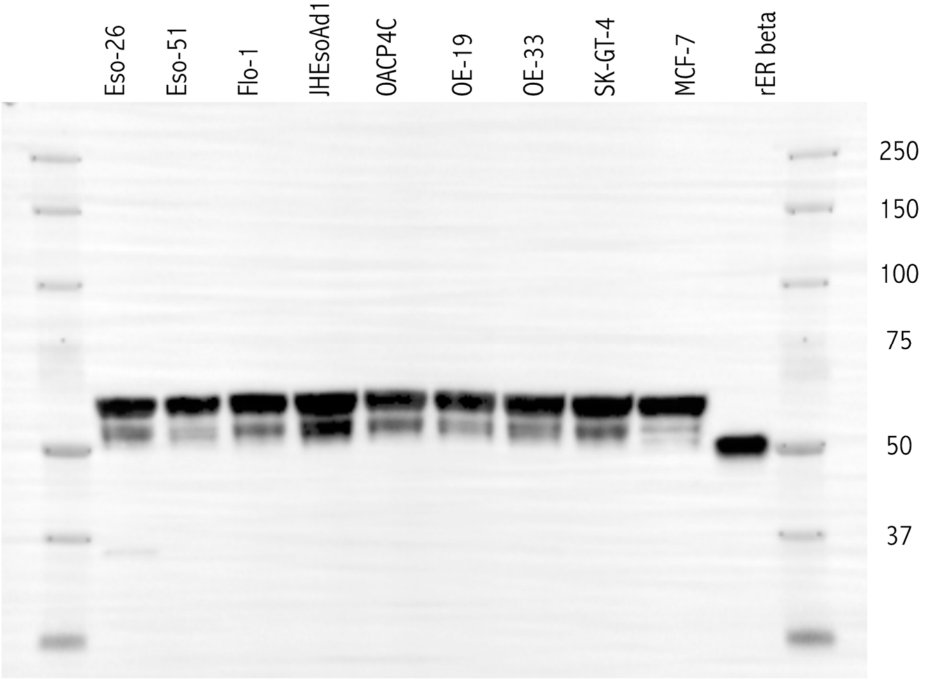

Figure S3. ERβ Western blot image presented in Figure 1B.

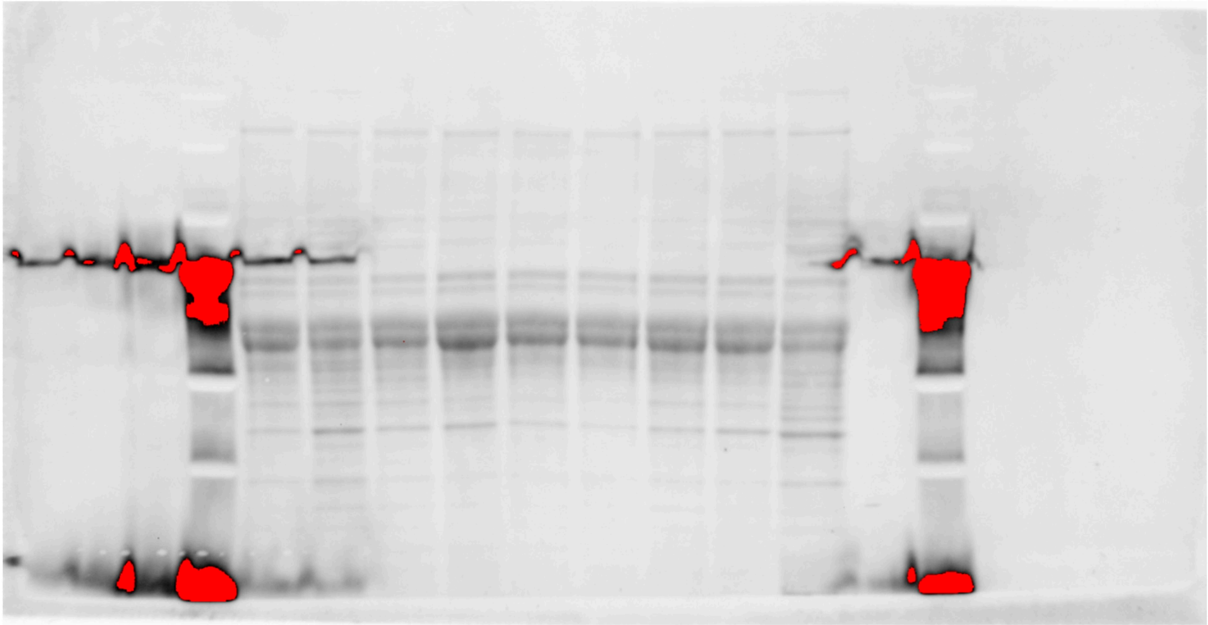

Figure S4. Total protein load image associated with Figure S3.

Table S3. Quantification data associated with ERβ blot presented in Figure 1B.

Serial Number	Net Intensity	Sum Intensity	Mean Intensity	Bkgnd. Intensity	Interior Area (pixels)	**Cell line**	**Normalisation Factor**
**ERβ western blot quantification**							
**Protein load normalisation**							
1	2404203.88	425908712	62096.8672	61807.4297	2160	Eso-26	1.30879791
2	2583395.88	426087904	62121.5117	61807.4297	2160	Eso-51	1.42622134
3	1815419.75	425319928	62028.4844	61807.4297	2160	Flo-1	0.94500557
4	2299547.75	425804056	62078.5664	61807.4297	2160	JHEsoAd1	1.24171905
5	1484987.75	424989496	61985.7109	61807.4297	2160	OACP4C	0.75446253
6	1200627.75	424705136	61946.5898	61807.4297	2160	OE-19	0.59765574
7	1747459.75	425251968	62021.8867	61807.4297	2160	OE-33	0.90505549
8	1727251.75	425231760	62027.8164	61807.4297	2160	SK-GT-4	0.89325366
**Quantification**							
**ER beta 59 kDa species**							

Serial Number	ROI Type	Net Intensity	Sum Intensity	Mean Intensity	Bkgnd. Intensity	Interior Area (pixels)	**Cell line**	**Normalised expression**
1	Rect	10019864	10073403	30525.4648	162.238281	330	Eso-26	7655776.27
2	Rect	9130841	9184380	27831.4531	162.238281	330	Eso-51	6402120.6
3	Rect	11055988	11109527	33665.2344	162.238281	330	Flo-1	11699389.3
4	Rect	12439573	12493112	37857.9141	162.238281	330	JHEso Ad1	10018025.4
5	Rect	9373757	9427296	28567.5625	162.238281	330	OACP 4C	12424416.8
6	Rect	9204273	9257812	28053.9766	162.238281	330	OE-19	15400626.9
7	Rect	10235905	10289444	31180.1328	162.238281	330	OE-33	11309698.8
8	Rect	11740353	11793892	35739.0664	162.238281	330	SK-GT-4	13143358.4

ER beta 54 kDa species

Serial Number	ROI Type	Net Intensity	Sum Intensity	Mean Intensity	Bkgnd. Intensity	Interior Area (pixels)	**Cell line**	**Normalised expression**
10	Rect	4730130	4783668.5	14495.9648	162.238281	330	Eso-26	3614102.65
11	Rect	2787205.75	2840744.5	8608.31641	162.238281	330	Eso-51	1954258.91
12	Rect	4817010	4870548.5	14759.2383	162.238281	330	Flo-1	5097335.06
13	Rect	8518917	8572456	25977.1406	162.238281	330	JHEso Ad1	6860583.34
14	Rect	5079654	5133192.5	15555.1289	162.238281	330	OACP 4C	6732811.47
15	Rect	3616365.5	3669904.25	11120.9219	162.238281	330	OE-19	6050917.4
16	Rect	5017477.5	5071016	15366.7148	162.238281	330	OE-33	5543834.09
17	Rect	5870545.5	5924084	17951.7695	162.238281	330	SK-GT-4	6572092.27

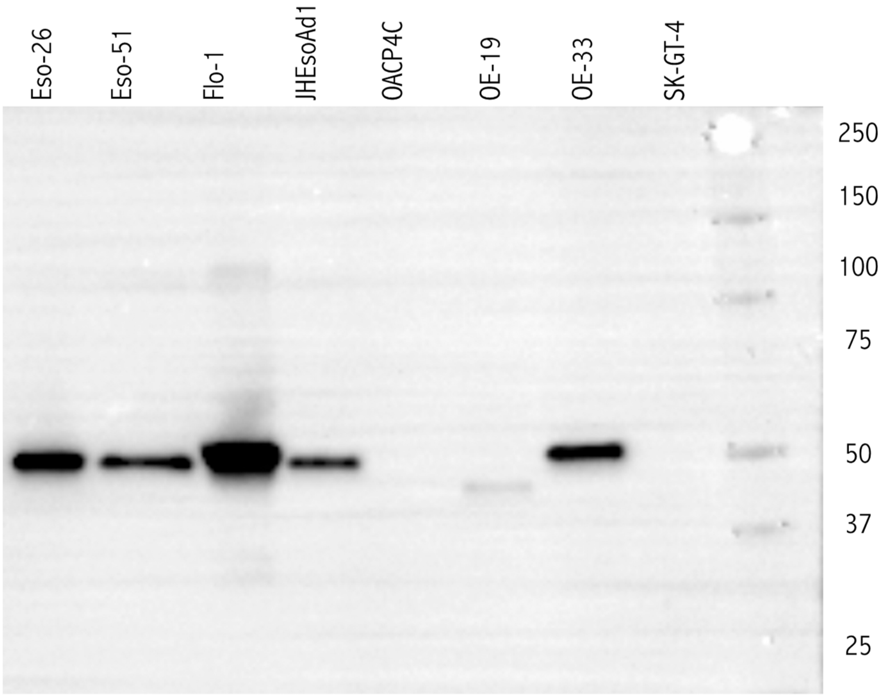

Figure S5. p53 Western blot image presented in Figure 4.

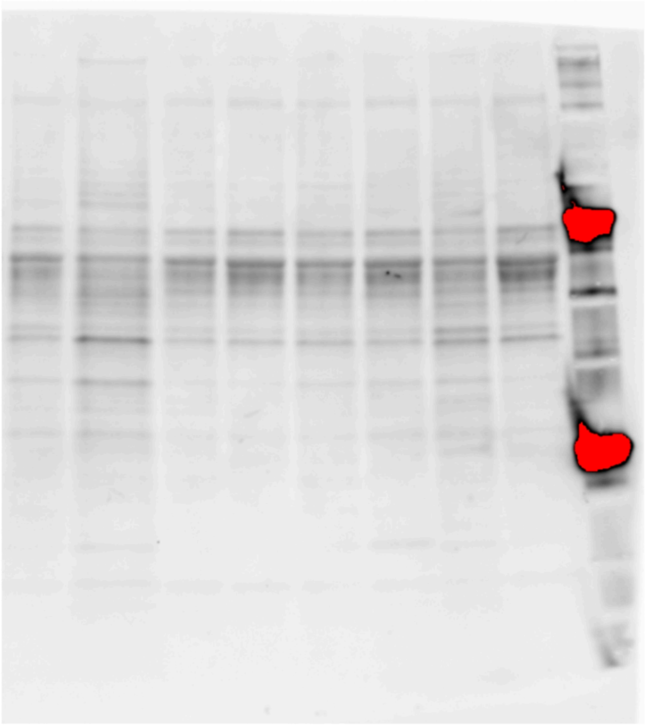

Figure S6. Total protein load image associated with Figure S5.

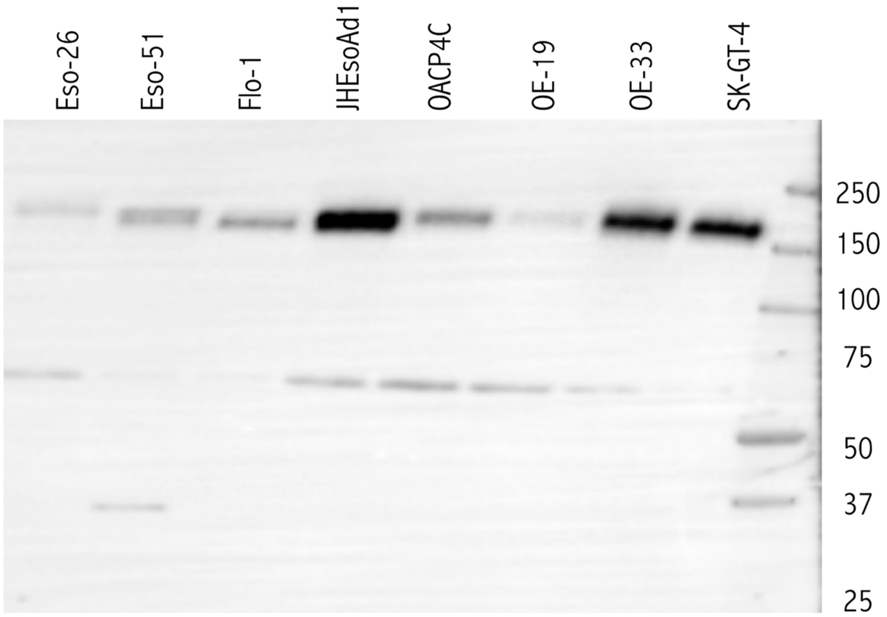

Figure S7. EGFR Western blot image presented in Figure 4.

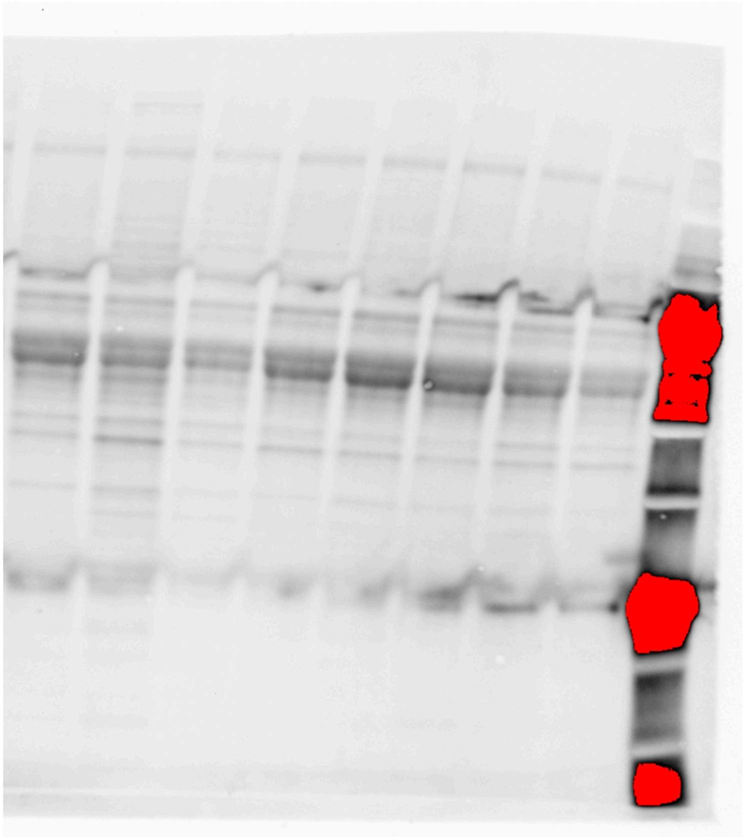

Figure S8. Total protein load image associated with Figure S7.

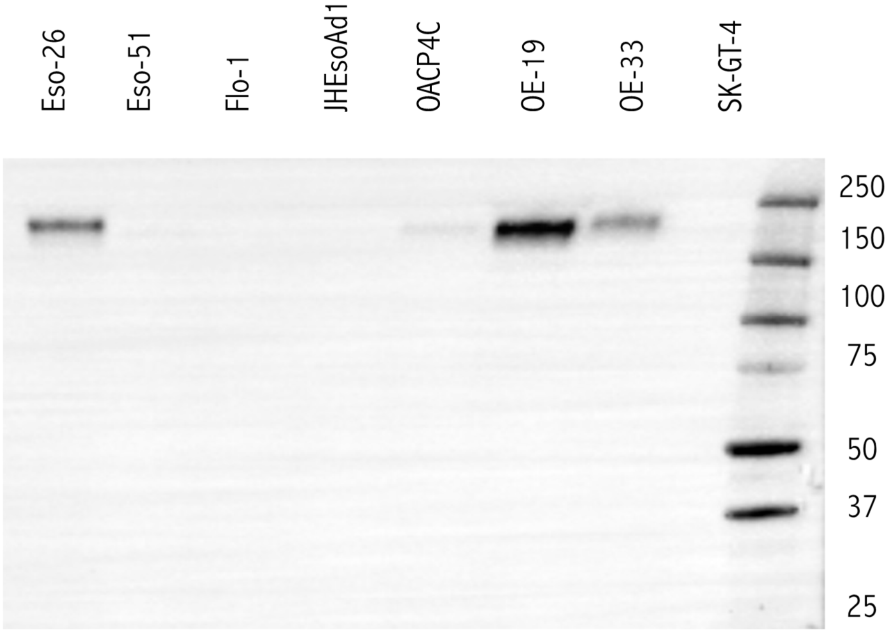

Figure S9. HER2 Western blot image presented in Figure 4.

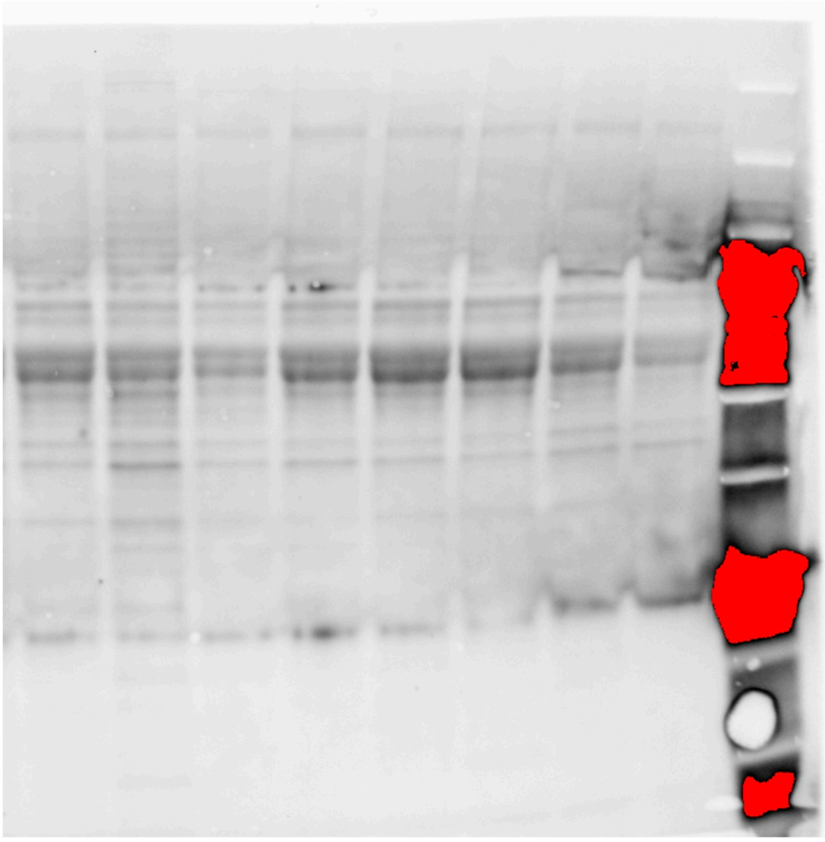

Figure S10. Total protein load image associated with Figure S9.

Table S4. Quantification data associated with p53 blot presented in Figure 4.

p53 western blot normalisation

Protein load normalisation

Serial Number	Net Intensity	Sum Intensity	Mean Intensity	Bkgnd. Intensity	Interior Area (pixels)	**Lysate**	**Normalisation (NET)**	**Normalisation (SUM)**	**Normalisation (MEAN)**
9	1,420,188.00	2,731,231.00	679.918098	326.373662	2490	16 Eso-26	0.93578961	0.96555	0.96555
10	1,618,194.00	2,929,237.00	729.210107	326.373662	2490	16 Eso-51	1.06625963	1.03554946	1.03554946
11	1,127,222.00	2,438,265.00	606.986557	326.373662	2490	16 Flo-1	0.74274859	0.8619801	0.8619801
12	1,260,737.00	2,571,780.00	640.224048	326.373662	2490	16 JHEso	0.83072423	0.90918058	0.90918058
13	1,224,792.00	2,535,835.00	631.275828	326.373662	2490	16 OAC	0.80703937	0.89647323	0.89647323
14	1,627,776.00	2,938,819.00	731.595469	326.373662	2490	16 OE-19	1.0725734	1.0389369	1.0389369
15	1,923,761.00	3,234,804.00	805.278566	326.373662	2490	16 OE-33	1.26760369	1.14357409	1.14357409
16	1,938,418.00	3,249,461.00	808.927309	326.373662	2490	16 SKGT4	1.27726148	1.14875566	1.14875566

Quantification

**p53** Serial Number	ROI Type	Net Intensity	Sum Intensity	Mean Intensity	Bkgnd. Intensity	Interior Area (pixels)	**Lysate**	**Normalised (Net)**	**Normalised (Sum)**	**Raw (Net)**	**Raw (Sum)**
9	Rect	1,083,532.00	1,085,444.00	6,167.30	10.863636	176	16 Eso-26	1157879.92	1122191.5	1083532	1085444
10	Rect	518,184.00	520,096.00	2,955.09	10.863636	176	16 Eso-51	485982.95	500395.222	518184	520096
11	Rect	2,995,984.00	2,997,896.00	17,033.50	10.863636	176	16 Flo-1	4033644.81	3475699.74	2995984	2997896

12	Rect	233,116.00	235,028.00	1,335.39	10.863636	176	16 JHEso	280617.792	256402.31	233116	235028
13	Rect	644	2,556.00	14.522727	10.863636	176	16 OAC	797.978419	718.370586	644	2556
14	Rect	52	1,964.00	11.159091	10.863636	176	16 OE-19	48.4815306	50.0511627	52	1964
15	Rect	468,724.00	470,636.00	2,674.07	10.863636	176	16 OE-33	369771.721	409876.374	468724	470636
16	Rect	-212	1,700.00	9.659091	10.863636	176	16 SKGT4	-165.98011	-184.54751	-212	1700

Table S5. Quantification data associated with EGFR blot presented in Figure 4.

EGFR western quantification

Protein load normalisation

Serial Number	Net Intensity	Sum Intensity	Mean Intensity	Bkgnd. Intensity	Interior Area (pixels)	**Lysate**	**Normalisation (NET)**	**Normalisation (SUM)**	**Normalisation (MEAN)**
9	590,745.00	3,284,737.00	643.561324	527.819749	5104	16 Eso-26	0.79062947	0.95453933	0.95453933
10	788,827.00	3,482,819.00	682.370494	527.819749	5104	16 Eso-51	1.0557345	1.01210164	1.01210164
11	543,965.00	3,237,957.00	634.395964	527.819749	5104	16 Flo-1	0.72802099	0.94094514	0.94094514
12	690,558.00	3,384,550.00	663.117163	527.819749	5104	16 JHEso	0.9242152	0.98354483	0.98354483
13	519,204.00	3,213,196.00	629.544671	527.819749	5104	16 OAC	0.69488186	0.93374963	0.93374963
14	728,791.00	3,422,783.00	670.607955	527.819749	5104	16 OE-19	0.97538472	0.99465528	0.99465528
15	1,044,035.00	3,738,027.00	732.372061	527.819749	5104	16 OE-33	1.39729467	1.08626468	1.08626468
16	1,071,340.00	3,765,332.00	737.721787	527.819749	5104	16 SKGT4	1.43383859	1.09419947	1.09419947

Quantification

EGFR (185 kD)

Serial Number	ROI Type	Net Intensity	Sum Intensity	Mean Intensity	Bkgnd. Intensity	Interior Area (pixels)	**Lysate**	**Normalised (Net)**	**Normalised (Sum)**	**Raw (Net)**	**Raw (Sum)**
9	Rect	20,300.00	52,912.00	229.056277	141.177489	231	16 Eso-26	25675.7441	21266.8031	20300	52912
10	Rect	254,164.00	286,776.00	1,241.45	141.177489	231	16 Eso-51	240746.135	251124.975	254164	286776
11	Rect	213,140.00	245,752.00	1,063.86	141.177489	231	16 Flo-1	292766.283	226516.926	213140	245752
12	Rect	3,197,056.00	3,229,668.00	13,981.25	141.177489	231	16 JHEso	3459211.67	3250544.26	3197056	3229668
13	Rect	755,060.00	787,672.00	3,409.84	141.177489	231	16 OAC	1086601.97	808632.181	755060	787672
14	Rect	154,092.00	186,704.00	808.242424	141.177489	231	16 OE-19	157980.741	154920.004	154092	186704
15	Rect	4,630,944.00	4,663,556.00	20,188.55	141.177489	231	16 OE-33	3314221.47	4263181.97	4630944	4663556
16	Rect	4,123,332.00	4,155,944.00	17,991.10	141.177489	231	16 SKGT4	2875729.54	3768354.96	4123332	4155944

Table S6. Quantification data associated with HER2 blot presented in Figure 4.

**HER2 western quantification**									
**Protein load normalisation**									
Serial Number	Net Intensity	Sum Intensity	Mean Intensity	Bkgnd. Intensity	Interior Area (pixels)	**Lysate**	**Normalisation (NET)**	**Normalisation (SUM)**	**Normalisation (MEAN)**
9	1,503,320.00	3,966,374.00	896.558318	556.748192	4424	16 Eso-26	0.90043513	0.95977634	0.95977634
10	1,815,566.00	4,278,620.00	967.138336	556.748192	4424	16 Eso-51	1.08745936	1.03533309	1.03533309
11	1,252,058.00	3,715,112.00	839.76311	556.748192	4424	16 Flo-1	0.74993814	0.8989764	0.8989764
12	1,577,772.00	4,040,826.00	913.387432	556.748192	4424	16 JHEso	0.94502922	0.97779211	0.97792111
13	2,011,329.00	4,474,383.00	1,011.39	556.748192	4424	16 OAC	1.20471442	1.08270349	1.08270349

14	1,925,760.00	4,388,814.00	992.046564	556.748192	4424		16 OE-19	1.15346164	1.06199765	1.06199765	
15	1,901,823.00	4,364,877.00	986.63585	556.748192	4424		16 OE-33	1.13912423	1.05620541	1.05620541	
16	1,368,759.00	3,831,813.00	866.142179	556.748192	4424		16 SKGT4	0.81983788	0.92721551	0.92721551	

Quantification

HER2 200 kD

Serial Number	ROI Type	Net Intensity	Sum Intensity	Mean Intensity	Bkgnd. Intensity	Interior Area (pixels)	**Lysate**	**Normalised (Net)**	**Normalised (Sum)**	**Raw (Net)**	**Raw (Sum)**
9	Rect	478,452.00	481,812.00	2,190.05	15.272727	220	16 Eso-26	531356.437	498503.639	478452	481812
10	Rect	4,200.00	7,560.00	34.363636	15.272727	220	16 Eso-51	3862.21331	4056.66546	4200	7560
11	Rect	2,748.00	6,108.00	27.763636	15.272727	220	16 Flo-1	3664.30224	3056.80995	2748	6108
12	Rect	1,952.00	5,312.00	24.145455	15.272727	220	16 JHEso	2065.5446	1996.33437	1952	5312
13	Rect	44,920.00	48,280.00	219.454545	15.272727	220	16 OAC	37286.8452	41488.7368	44920	48280
14	Rect	4,544,412.00	4,547,772.00	20,671.69	15.272727	220	16 OE-19	3939803.33	4279116.82	4544412	4547772
15	Rect	620,356.00	623,716.00	2,835.07	15.272727	220	16 OE-33	544590.297	587344.083	620356	623716
16	Rect	2,236.00	5,596.00	25.436364	15.272727	220	16 SKGT4	2727.36849	2411.52136	2236	5596

Receptor expression and treatment response experiments

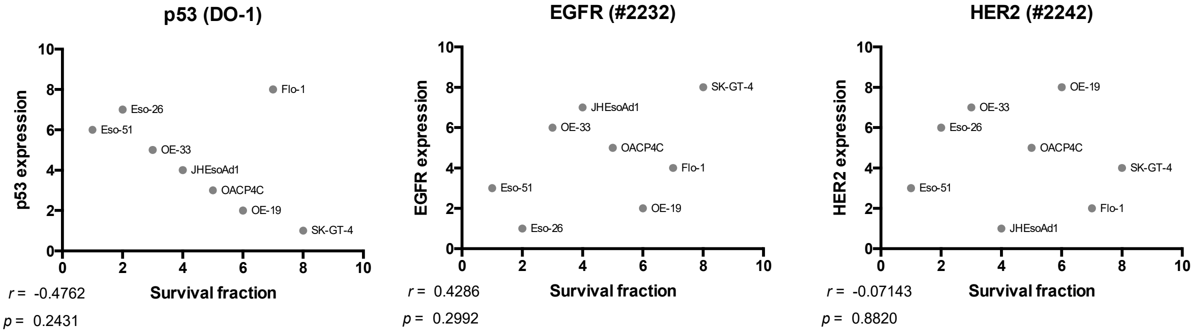

Figure S11. Scatterplots of rank showing relationship between survival fraction and the expression of p53, EGFR, and HER2, in all cell lines.

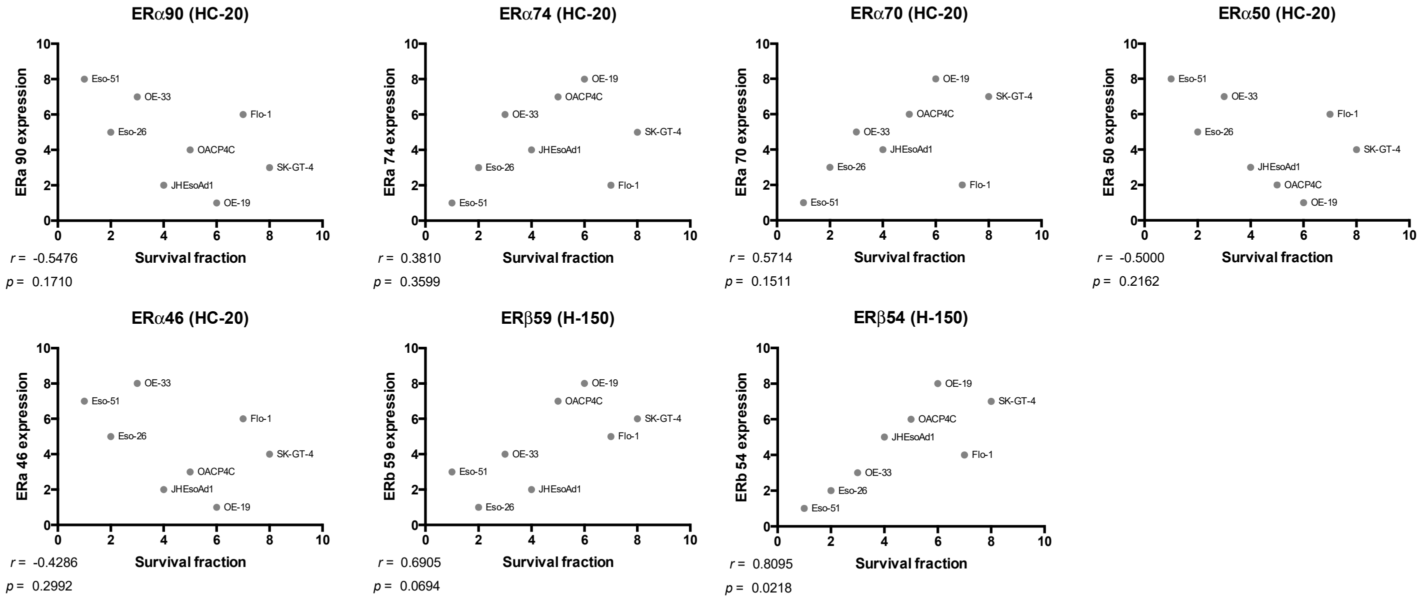

Figure S12. Scatterplots of ranks showing relationship between survival fraction and the expression of ERα and ERβ species, in all cell lines.

Table S7. ***TP53*** **mutations in oesophageal adenocarcinoma cell lines, and their consequences.** c., CDNA sequence; p., protein (amino acid) sequence; *, nonsense (noncoding) substitution; ins, nucleotide insertion; fs, frameshift; SIFT, Sorting Intolerant From Tolerant (program that predicts the effects of amino acid substitutions upon protein function) [8]; COSMIC, Catalogue of Somatic Mutations in Cancer [9]; IARC, International Agency for Research on Cancer *TP53* mutation database [10]; na, not available. Reproduced with permission from [11].

Cell line.	*TP53* DNA Mutation	p53 Protein Mutation	p53 Domain	Phenotype	Transcriptional Activity Class	SIFT Prediction	Reference
Eso-26	c.742C>T	p.R248W	DNA binding domain	Missense substitution	Non-functional	Deleterious	COSMIC, IARC
Eso-51	c.524G>A	p.R175H	DNA binding domain	Missense substitution	Non-functional	Deleterious	COSMIC, IARC
Flo-1	c.830G>T	p.C277F	DNA binding domain	Missense substitution	Non-functional	Deleterious	COSMIC, IARC
JH-EsoAd1	c.797G>A	p.G266E	DNA binding domain	Missense substitution	Non-functional	Deleterious	[3], IARC

OACP4C	c.574C>T	p.Q192*	DNA binding domain	Nonsense substitution (? Truncation)	na	na	COSMIC, IARC
OE-19	c.927_928insA	p.N310fs*27	Homooligomerisation domain	Frameshift insertion			COSMIC
	c.929_930ins1	p.?	Homooligomerisation domain	Frameshift insertion	na	na	IARC
OE-33	c.404G>A	p.C135Y	DNA binding domain	Missense substitution	Non-functional	Deleterious	COSMIC, IARC
SK-GT-4	c.298C>T	p.Q100*	DNA binding domain	Nonsense substitution (? Truncation)	na	na	COSMIC, IARC
	c.524G>A	p.R175H	DNA binding domain	Missense substitution	Non-functional	Deleterious	IARC

References

1. Boonstra, J. J.; van Marion, R.; Beer, D.G.; Lin, L.; Chaves, P.; Ribeiro, C.; Pereira, A.D.; Roque, L.; Darnton, S.J.; Altorki, N.K. et al., Verification and unmasking of widely used human esophageal adenocarcinoma cell lines. *J Natl Cancer Inst*, **2010,** *102*, 271–274.
2. Hughes, S. J., Nambu, Y.; Soldes, O.S.; Hamstra, D.; Rehemtulla, A.; Iannettoni, M.D.; Orringer, M.B.; Beer,D.G. Fas/APO-1 (CD95) is not translocated to the cell membrane in esophageal adenocarcinoma. *Cancer Res*, **1997**, *57*, 5571–5578.
3. Alvarez, H.; Koorstra, J.B.; Hong, S.M.; Boonstra, J.J.; Dinjens, W.N.; Foratiere, A.A.; Wu, T.T.; Montgomery, E.; Eshleman, J.R.; Maitra, A. Establishment and characterization of a bona fide Barrett esophagus-associated adenocarcinoma cell line. *Cancer Biol Ther*, **2008**, *7*, 1753–1755.
4. de Both, N. J.; Wijnhoven, B.P.L.; Sleddens, H.F.B.M; Tilanus, H.W.; Dinjens, W.N.M. Establishment of cell lines from adenocarcinomas of the esophagus and gastric cardia growing in vivo and in vitro. *Virchows Archiv*, **2001**, *438*, 451–456.
5. Rockett, J. C.; Larkin, K.; Darnton, S.J.; Morris, A.G.; Matthews, H.R. Five newly established oesophageal carcinoma cell lines: phenotypic and immunological characterization. *Br J Cancer*, **1997**, *75*, 258–263.
6. Altorki, N.; Schwartz, G.K.; Blundell, M.; Davis, B.M.; Kelsen, D.P.;Albino, A.P. Characterization of cell lines established from human gastric-esophageal adenocarcinomas. Biologic phenotype and invasion potential. *Cancer,* **1993**, *72*, 649–657.
7. Soule, H. D.; Vazguez, J.; Long, A.; Albert, S.; Brennan, M. A human cell line from a pleural effusion derived from a breast carcinoma. *J Natl Cancer Inst*, **1973**, *51*, 1409–1416.
8. Sim, N.L.; Kumar, P.; Hu, J.; Henikoff, S.; Schneider, G.; Ng, P.C. SIFT web server: predicting effects of amino acid substitutions on proteins. *Nucleic Acids Research*, **2012**, *40*, 452–457.
9. Forbes, S. A.; Beare, D.; Boutselakis, H.; Bamford, S.; Bindal, N.; Tate, J.;. Cole, C.G.; Ward, S.; Dawson, E.; Ponting, L. et al., COSMIC: somatic cancer genetics at high-resolution. *Nucleic Acids Res*, **2017**, *45*, 777–783.
10. Bouaoun, L.; Sonkin, D.; Ardin, M.; Hollstein, M.; Byrnes, G.; Zavadil, J.; Olivier, M. TP53 Variations in Human Cancers: New Lessons from the IARC TP53 Database and Genomics Data. *Hum Mutat*, **2016**, *37*, 865–876.
11. Butz, F.; Eichelmann, A.-K.; Mayne, G.C.; Wang, T.; Bastian, I.; Chiam, K.; Marri, S.; Sykes, P.J.; Wijnhoven, B.P.; Toxopeus,E. et al. MicroRNA Profiling in Oesophageal Adenocarcinoma Cell Lines and Patient Serum Samples Reveals a Role for miR-451a in Radiation Resistance. *International Journal of Molecular Sciences*. **2020**; *21*, 8898.
